# Modified translationally controlled tumor protein-derived protein transduction domain enhances nasal delivery of exendin-4 as shown with insulin

**DOI:** 10.1080/10717544.2018.1491653

**Published:** 2018-07-25

**Authors:** Hae-Duck Bae, Moonhee Kim, Joohyun Lee, Kyunglim Lee

**Affiliations:** Graduate School of Pharmaceutical Sciences, College of Pharmacy, Ewha Womans University, Seoul, South Korea

**Keywords:** Drug delivery, exendin-4, intranasal absorption, protein transduction domain, translationally controlled tumor protein

## Abstract

Protein transduction domains (PTDs) have been shown to promote the delivery of therapeutic proteins or peptides into the living cells. In a previous study, we showed that the double mutant of TCTP-PTD 13, TCTP-PTD 13M2, was more effective in the delivery of insulin than the wild-type TCTP-PTD 13. In this study, we applied this approach to the nasal delivery of a different peptide, exendin-4, using as carriers, several modified TCTP-PTDs, such as TCTP-PTD 13M1, 13M2, and 13M3. Nasal co-administration of TCTP-PTD 13M2 with exendin-4 showed the highest exendin-4 uptake among the three analogs in normal rats, and also decreased blood glucose levels by 43.3% compared with that of exendin-4 alone and by 18.6% compared with that of exendin-4 plus TCTP-PTD 13 in diabetic mice. We also designed an additional covalently linked conjugate of TCTP-PTD 13M2 and exendin-4 and evaluated its hypoglycemic effect after subcutaneous or intranasal delivery. Subcutaneous administration of exendin-4 that its C-terminus is covalently linked to TCTP-PTD 13M2 showed hypoglycemic effect of 42.2% compared to that in untreated group, whereas intranasal delivery was not successful in diabetic mice. We conclude that a simple mixing TCTP-PTD 13M2 with peptide/protein drugs can be potentially a generally applicable approach for intranasal delivery into animals.

## Introduction

Nasal drug delivery has advantages such as rapid absorption, self-administration, non-invasiveness, and avoidance of the hepatic first-pass effect (Chien & Chang, 1987). However, the bioavailability of peptides and proteins administered intranasally is often low. Attempts to overcome this have led to the use of absorption enhancers (Davis & Illum, 2003) and nanosystems (Battaglia et al., 2018), but proved toxic to the nasal mucosa. This led to the use of protein transduction domains (PTDs) for peptide/protein drug delivery by others (Khafagy El et al., 2010) and by our group (Bae & Lee, 2013; Bae et al., 2018).

PTDs are short peptide fragments of proteins, consisting of 8-30 amino acids, that proved useful to deliver various cargoes across cell membrane lipid bilayer. Two approaches have been used to link the PTD to the therapeutic drug: the fusion type, which involved covalent linkage of the cargo and PTD, and the mixture type, which involved using simple mixtures of the two (Gros et al., 2006).

Previously, we reported that translationally controlled tumor protein (TCTP) has a 10-amino acid- long PTD at its NH_2_-terminus (Kim et al., 2011a). In attempts to increase the penetrating activity of natural TCTP-PTD, we prepared several analogous mutants and examined their ability to increase to serve as transport vehicles for peptide/protein into cells. We found that TCTP-PTD 13 (MIIFRALISHKK) exhibited increased ability to penetrate cell membrane with no cytotoxic side effects (Kim et al., 2011b). Recently, we reported that the co-administration of mixtures of insulin with TCTP-PTD 13 or its mutant analogs efficiently delivered bioavailable insulin into the nasal mucosal membrane of both normal and diabetic rats without causing nasal membrane damage (Bae et al., 2018). In this study, we are reporting on the general applicability of this approach, employing another peptide, exendin-4, a 39 amino acids glucagon-like peptide-1 (GLP-1) anti-diabetic agent, and its intranasal delivery using a variety of modified TCTP-PTDs as vehicles for intranasal delivery. We examined whether TCTP-PTD 13 and its analogs increased nasal exendin-4 absorption in normal rats and the hypoglycemic effect of exendin-4 in diabetic mice and investigated the toxicity of TCTP-PTD analog mixtures intranasally co-administered with exendin-4. We also prepared TCTP-PTD analogs covalently attached to N-terminal or C-terminal of exendin-4 and compared their blood glucose lowering effect with those of simple mixtures of TCTP-PTD 13 analogs and exendin-4.

## Materials and methods

### Materials and animals

Exendin-4 was purchased from Enzo Life Science, Inc. (Farmingdale, NY). Sodium taurodeoxycholate and d-glucose were obtained from Sigma-Aldrich (St. Louis, MO). TCTP-PTD analogs and chimeric PTD-exendin-4 peptides were synthesized by A&PEP, Inc. (Cheongju, South Korea). All TCTP-PTD analogs were acetylated at the N-terminus while their C-termini were protected by amidation. The purity of the peptides verified by high-performance liquid chromatography was >95%. All the chemicals used in this study were of analytical grade.

Male Wistar rats and C57BL/6 *db/db* mice at 6 weeks of age were purchased from the Young Bio Co., Ltd. (Seongnam, South Korea). They were housed in a temperature and humidity controlled room with a 12:12 h light–dark cycle and were allowed food and water. All animal procedures were approved by Ewha Womans University’s Institutional Animal Care and Use Committee (approval ID: 14-095).

### Preparation of exendin-4/PTD mixtures

To evaluate the nasal pharmacokinetic (PK) profile of exendin-4 in rats, we simply mixed it with the PTD (1:2 molar ratio) (Bae et al., 2018). One hundred microliters of exendin-4 (200 µM) were mixed with 100 µL of PTD (400 µM) in 10 mM phosphate buffer (pH 6.4). To evaluate biological activities of exendin-4 in type 2 *db/db* mice, exendin-4 (10 μM) was mixed with PTD (20 μM) in 10 mM phosphate buffer (pH 6.4) for nasal administration. The exendin-4/PTD mixtures were visually inspected to confirm cloudiness (turbidity) and precipitation. All exendin-4/PTD mixtures were clear.

### Pharmacokinetics studies in rats

Rats (180–200 g) were fasted overnight with free access to water. The animals anesthetized by intraperitoneal (i.p.) injection of sodium pentobarbital (60 mg/kg) and placed in the supine position. To evaluate absorption of the exendin-4 through the nasal mucosa, exendin-4 with or without PTD solution (dose of exendin-4:30 µg/kg) was administered into the right nostril of the rats using a pipette. To assess the relative bioavailability (BA), exendin-4 solution was administered by subcutaneous (s.c.) route at a dose of 10 μg/kg. Hundred microliters of blood samples were collected from the rat tail 5, 10, 20, 30, 60, 90, 120, and 180 min after dosing. Plasma samples were obtained after centrifugation at 4000×*g* for 25 min.

The relative BA values of nasally administered exendin-4 were determined relative to the s.c. injection. The maximum plasma concentration (*C*_max_) and time to reach *C*_max_ (*T*_max_) were directly determined from the plasma concentration–time profiles. The area under the concentration–time curve (AUC) from 0 to 180 min (AUC_0–180 min_) was calculated by use of the trapezoid rule. Relative BA was calculated to as follows:

$$\text{BA}\;\left(\%\right)\;=\;\left({\text{AUC}}_{\text{nasal}}\times {\;\text{Dose}}_{s.c.}\right)/\left({\text{AUC}}_{s.c.}\times {\;\text{Dose}}_{\text{nasal}}\right)\;\times \;100\%$$

### Measurement of plasma exendin-4 by ELISA

A 96 well high binding plate (Costar, Corning Inc., Corning, NY) was coated with 100 µL/well of the monoclonal anti-exendin-4 as a capture antibody (1:500; BioPorto Co.; ABS 033-10) in 50 mM sodium carbonate, pH 9.6 by incubating the plate overnight at 4 °C. After washing three times in washing buffer (50 mM Tris, 0.14 M NaCl at pH 7.4 with 0.05% (v/v) Tween 20), the plates were blocked by adding 200 µL/well of blocking solution (50 mM Tris, 0.14 M NaCl at pH 7.4 with 1% (v/v) bovine serum albumin) and incubated for 30 min at room temperature, then washed an additional three times. The plates were then incubated with the standard of exendin-4 and samples at 100 µL/well for 2 h in an incubator shaker at 200 rpm and 21–23 °C. The standard was serially diluted within the range of 0.05–5 ng/mL, and the samples were diluted 5-fold in a specialized buffer (BUF037A; Bio-Rad, Hercules, CA) that reduce the difference between the sample and the standard curve. The plates were washed three times, and biotinylated anti-exendin-4 detection antibody (1:5000; BioPorto Co., Hellerup, Denmark; ABS 012-35) was added to the plates at 100 µL/well. After 1 h incubation, the plate was washed again three times and incubated with streptavidin and horseradish peroxidase (1:10,000; Sigma) for 30 min. The plates were washed an additional five times, and a color reaction was initiated by adding 100 µL 0.01% (v/v) 3,3′,5,5′-tetramethylbenzidine (TMB) solution (50 mM sodium acetate at pH 5.2, DMSO, with 30% (v/v) H_2_O_2_). The reaction mixture was terminated by adding 100 µL of 0.2 M H_2_SO_4_, and the absorbance was measured at 450 nm using a microplate reader.

### Hypoglycemic activity in type 2 diabetic mice

Type 2 diabetic male *db/db* mice (7–10 weeks old) were used for an i.p. glucose tolerance test (IPGTT) after nasal administration. After the mice were weighed, the mice were anesthetized by an i.p. injection of sodium pentobarbital (75 mg/kg). Prior to nasal administration, blood samples were taken from the mouse tail to record baseline blood glucose levels. At 30 min prior to the i.p. administration of glucose (1.2 g/kg), overnight fasted *db/db* mice were nasally administered exendin-4 or exendin-4/PTD mixtures (does of exendin-4:5 µg/kg) to the right nostril. The blood glucose levels were monitored using glucose meter (Accu-Chek, Roche Diagnostics, Seoul, South Korea). The blood glucose levels were monitored at −30, 0, 30, 60, 90, 120, and 180 min intervals.

### Lactate dehydrogenase (LDH) leakage in nasal fluid

The exendin-4/PTD mixtures prepared as described above were applied to the nostrils of anesthetized rats (dose of exendin-4, 30 µg/kg). Untreated rats served as negative controls. The positive control group was nasally administered to rats with 5% (w/v) sodium taurodeoxycholate. After 15 min, the nasal cavity was washed with 1 mL PBS using a micropipette. The washed solution was collected, and LDH activity in the wash solution was measured using a CytoTox-96 assay kit (Promega, Madison, WI) according to the manufacturer’s protocol. LDH leakage into the nasal fluid after the nasal administration of 5% (w/v) sodium taurodeoxycholate was defined as 100% leakage.

### Statistical analysis

Statistical analysis was determined by using Prism 5 software package (GraphPad Inc., La Jolla, CA). Statistical significances were determined using the Student’s t-test. For multiple comparisons, the significance of differences in mean values was evaluated using one-way analysis of variance (ANOVA) and Dunnett’s test. All error bars were expressed as the mean ± the standard error of the mean (SEM). The statistical significance was accepted at a value of *p* < .05.

## Results and discussion

### Designs of TCTP-PTD analogs

In a previous study, we constructed single mutant, A6L of TCTP-PTD 13 and double mutant, A6L, I8A of TCTP-PTD 13 and designated TCTP-PTD 13M1 and TCTP-PTD 13M2, respectively and demonstrated that TCTP-PTD 13M2 was found to be a useful vehicle for the delivery of insulin through the nasal membrane (Bae et al., 2018). In this study, we additionally designed a substitution S9Y of TCTP-PTD 13M2, called TCTP-PTD 13M3 (Table 1), because of its higher hydrophobicity than TCTP-PTD 13M2, might be better at cargo delivery than others. TCTP-PTD 13M3 did not form any precipitations in our experimental conditions, even though it is more hydrophobic than TCTP-PTD 13M2 (data not shown). The reason is considered that tyrosine has a good solubility in an aqueous solution due to its hydroxyl group even though tyrosine is more hydrophobic than serine.

**Table 1.** Peptides used in this study. The theoretical isoelectric point and molecular weight were obtained using tools on the ExPASy server.

Name	Sequence^a^	pI^b^	MW (Da)^c^
TCTP-PTD 13	MIIFRALISHKK	11.17	1456.86
TCTP-PTD 13M1	MIIFRLLISHKK	11.17	1498.94
TCTP-PTD 13M2	MIIFRLLASHKK	11.17	1456.86
TCTP-PTD 13M3	MIIFRLLAYHKK	10.29	1532.95

a The position of amino acid substitutions is indicated by shading (gray).

b Isoelectric point.

c Molecular weight.

### Pharmacokinetic studies in normal rats

To evaluate the ability of TCTP-PTD analogs to increase the exendin-4 that delivered through the nasal administration, we performed PKs studies in normal rats. We studied using the simple mixtures and fixed their amounts based on our previous studies (Bae et al., 2018).

**Figure 1.**
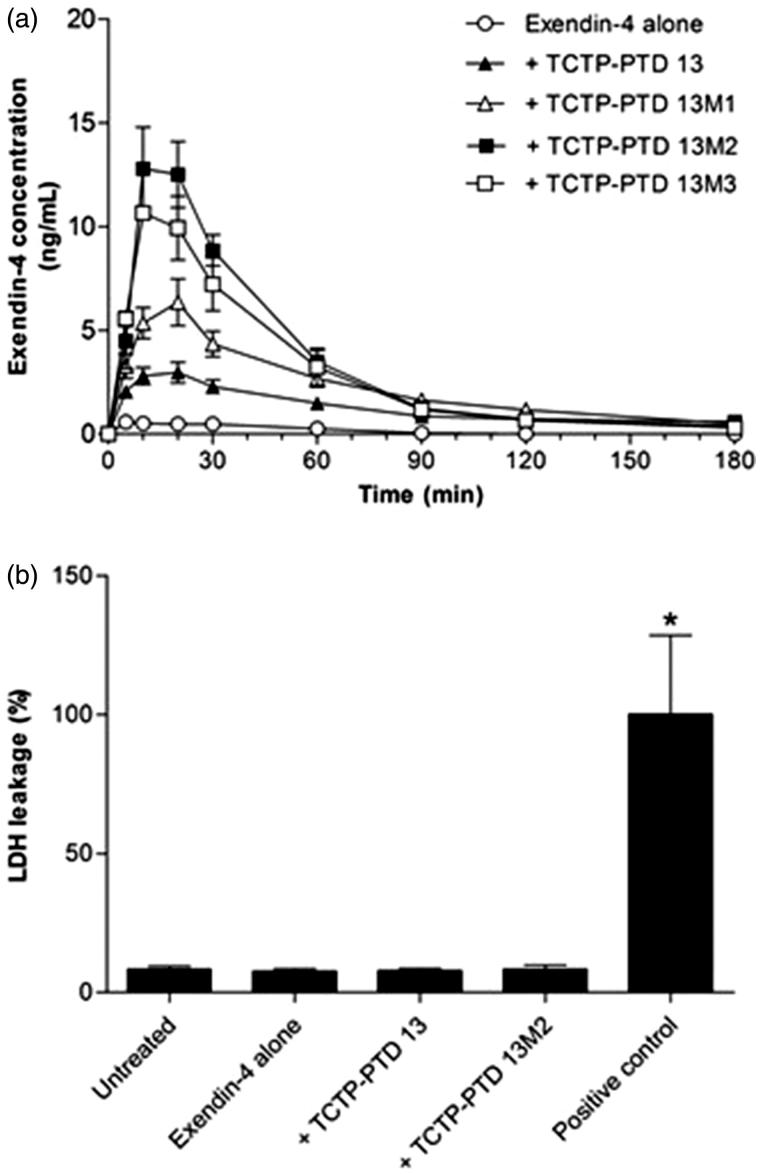
(a) Plasma concentration profiles of exendin-4 in normal rats following nasal administration of exendin-4 (30 µg/kg) with or without PTDs. Vertical bars indicate means ± SEM (*n* = 5–6). (b) LDH leakage in nasal fluid of normal rats following nasal administration of exendin-4 and exendin-4/PTD mixture. Five percent (w/v) sodium taurodeoxycholate was used as a positive control. The LDH leakage of positive control was set to 100%. Values are expressed as means ± SEM (*n* = 7). *p < .05 compared with the untreated group.

When 30 µg/kg of exendin-4 was administered alone (without PTDs) nasally, it was hardly detected in blood samples of normal rats, whereas exendin-4 was administered along with TCTP-PTD analogs there was detectable exendin-4 in blood, indicating it was absorbed. The order of absorption rates was: TCTP-PTD 13M2, TCTP-PTD 13M3, TCTP-PTD 13M1, and TCTP-PTD 13; the absorption of newly designed TCTP-PTD 13M3 was as effective as that of TCTP-PTD 13M2 (Figure 1(a)). The relative BAs of exendin-4 alone, and with TCTP-PTD 13, TCTP-PTD 13M1, TCTP-PTD 13M2, and TCTP-PTD 13M3, respectively were 1.6, 11.4, 20.7, 31.5, and 29.3%, compared to the absorption of exendin-4 administered by subcutaneous route at a dose of 10 μg/kg (Table 2). The PK parameters following nasal administration of exendin-4/PTD mixture in normal rats are also listed in Table 2. We found that intranasal absorption of exendin-4 plus TCTP-PTD 13M2 was the highest, as was the case before with insulin (Bae et al., 2018); and that of exendin-4 plus TCTP-PTD 13M3 was as high as that of TCTP-PTD 13M2, based on the values of *C*_max_, AUC, and BA. *T*_max_ of exendin-4 plus TCTP-PTD 13M2 was 16.7 min.

**Table 2.** Pharmacokinetic parameters following nasal administration of exendin-4/PTD mixture in Wistar rats.

Formulation	*T*_max_ (min)	*C*_max_ (% baseline)	AUC (ng/mL· min)	BA (%)
Exendin-4 alone	14 ± 2.4	0.7 ± 0.0	30.0 ± 5.5	1.6 ± 0.3
+ TCTP-PTD 13	16 ± 2.4	3.0 ± 0.5^a^	221.2 ± 36.9^a^	11.4 ± 1.9^a^
+ TCTP-PTD 13M1	20 ± 0.0	6.4 ± 1.1^a^	403.2 ± 66.6^a^	20.7 ± 3.7^a^
+ TCTP-PTD 13M2	16.7 ± 2.0	13.6 ± 1.8^a^^,^^b^	614.0 ± 77.0^a^^,^^b^	31.5 ± 4.0^a^^,^^b^
+ TCTP-PTD 13M3	16 ± 2.4	11.9 ± 1.0^a^^,^^b^	570.8 ± 93.0^a^^,^^b^	29.3 ± 4.8^a^^,^^b^

Values are expressed as means ± SEM (*n* = 5–6).

*T*_max_: time to reach maximum concentration *C*_max_; *C*_max_: maximum concentration; AUC: area under the curve; BA: relative bioavailability compared with s.c.; s.c.: subcutaneous

a Significantly different from nasal administration of exendin-4 at *p* < .05.

b Significantly different from nasal administration of exendin-4 plus TCTP-PTD 13 at *p* < .05.

The enhanced hydrophobicity of TCTP-PTD 13M3 resulted in an efficient intranasal delivery of exendin-4 but less efficient than that of TCTP-PTD 13M2, agreeing with a previous suggestion that the absorption of PTD may vary depending on the cargo (Maiolo et al., 2005; Bolhassani et al., 2017).

### LDH activity in nasal fluid of normal rats

We performed LDH assay to see whether the enhanced delivery of exendin-4 was caused by damage to the nasal mucous membrane following co-administration of TCTP-PTD analogs. When LDH leakage was set at 100% by the treatment of sodium taurodeoxycholate (positive control), there was no LDH leakage by co-administration with TCTP-PTD 13 and 13M2. These results indicated that nasal co-administration of TCTP-PTD 13M2 enhanced exendin-4 delivery without damaging nasal mucous membrane.

### Pharmacological studies in diabetic mice

**Figure 2.**
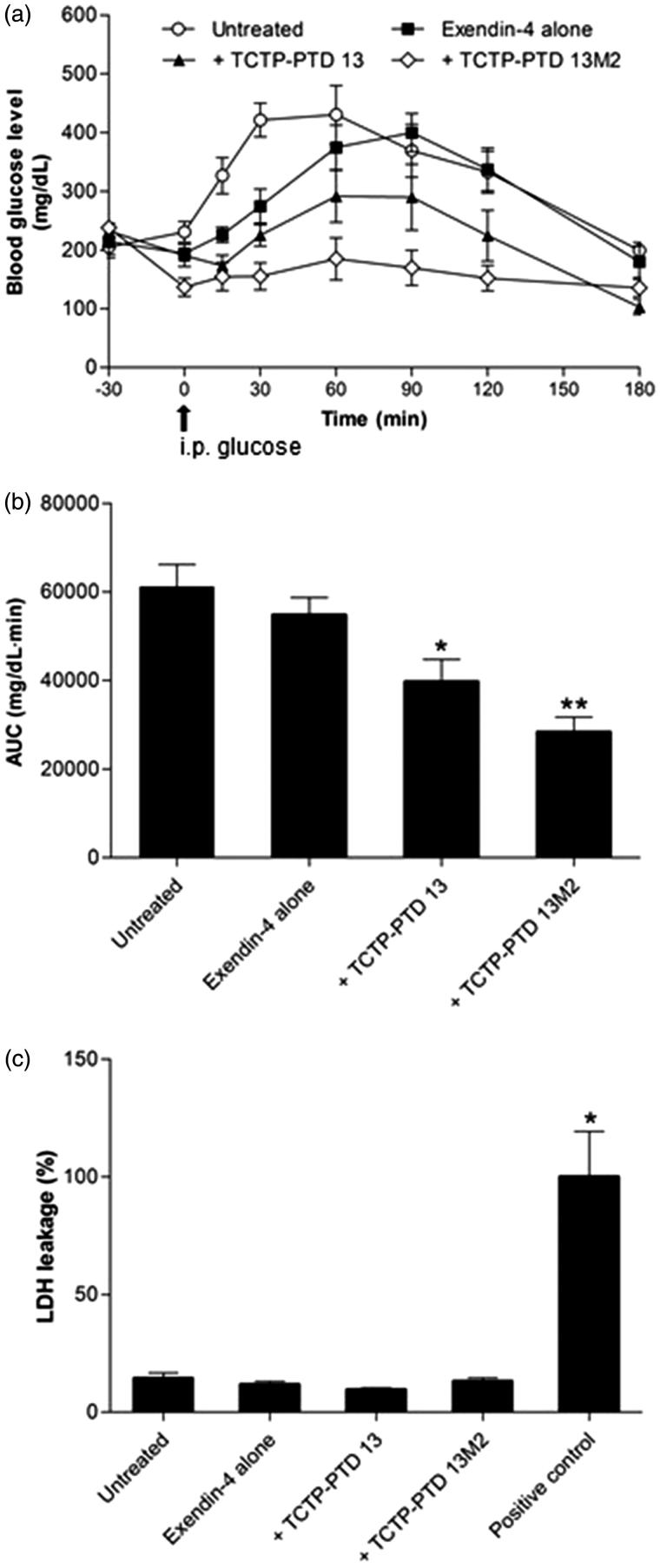
(a) Changes in blood glucose levels in type 2 *db/db* mice after nasal administration of exendin-4 and exendin-4/PTD mixtures at a dose of 5 μg/kg. The time of glucose challenge in type 2 *db/db* mice is marked by the arrow. (b) Changes in the glucose response are expressed as the AUC. Vertical bars indicate means ± SEM (*n* = 6–7). *p < .05 and **p < .01 compared with the exendin-4 alone. (c) LDH leakage in nasal fluid of type 2 *db/db* mice following nasal administration of exendin-4 and exendin-4/PTD mixtures. Nasal wash samples were collected at the end of the experiment and used LDH assay. Five % (w/v) sodium taurodeoxycholate was used as a positive control. The LDH leakage of positive control was set to 100%. Values are expressed as means ± SEM (*n* = 5–6). *p < .05 compared with the untreated group.

In order to confirm that exendin-4 delivered by TCTP-PTD is functional, we measured its hypoglycemic effect in the type 2 *db/db* mice. Thirty minutes before the glucose was administered, exendin-4 with or without TCTP- PTD 13 and TCTP- PTD 13M2 were intranasally administrated. We found that TCTP-PTD 13M2 was the best (Figure 2(a)). The AUC of exendin-4 alone, or with TCTP- PTD 13, and TCTP-PTD 13M2 were 54840, 39729, and 28390, respectively, and decreased by 10.2, 34.9, and 53.5%, respectively, compared with that in the untreated mice (Figure 2(b)). Consistent with the above results, exendin-4 plus TCTP-PTD 13M2 showed the strongest blood glucose lowering effect.

To check the nasal mucosal damage caused by nasal administration in diabetic mice, we performed LDH leakage assay in nasal wash fluids from each mice group. When the LDH leakage level of the positive control treated with sodium taurodeoxycholate was set at 100%, there was no LDH leakage in the co-administration groups of TCTP-PTD 13 and TCTP-PTD 13M2. These results demonstrate that exendin-4 delivery enhanced by the mixture with TCTP-PTD 13M2 was not due to damage of the nasal tissue in diabetic mice (Figure 2(c)). These results suggest that intranasal administration of exendin-4 plus TCTP-PTD 13M2 can effectively enhance hypoglycemic effects without toxicity in a mouse model of diabetes.

### Synthesis of PTD-exendin-4 linked peptides and pharmacological studies in diabetic mice

**Figure 3.**
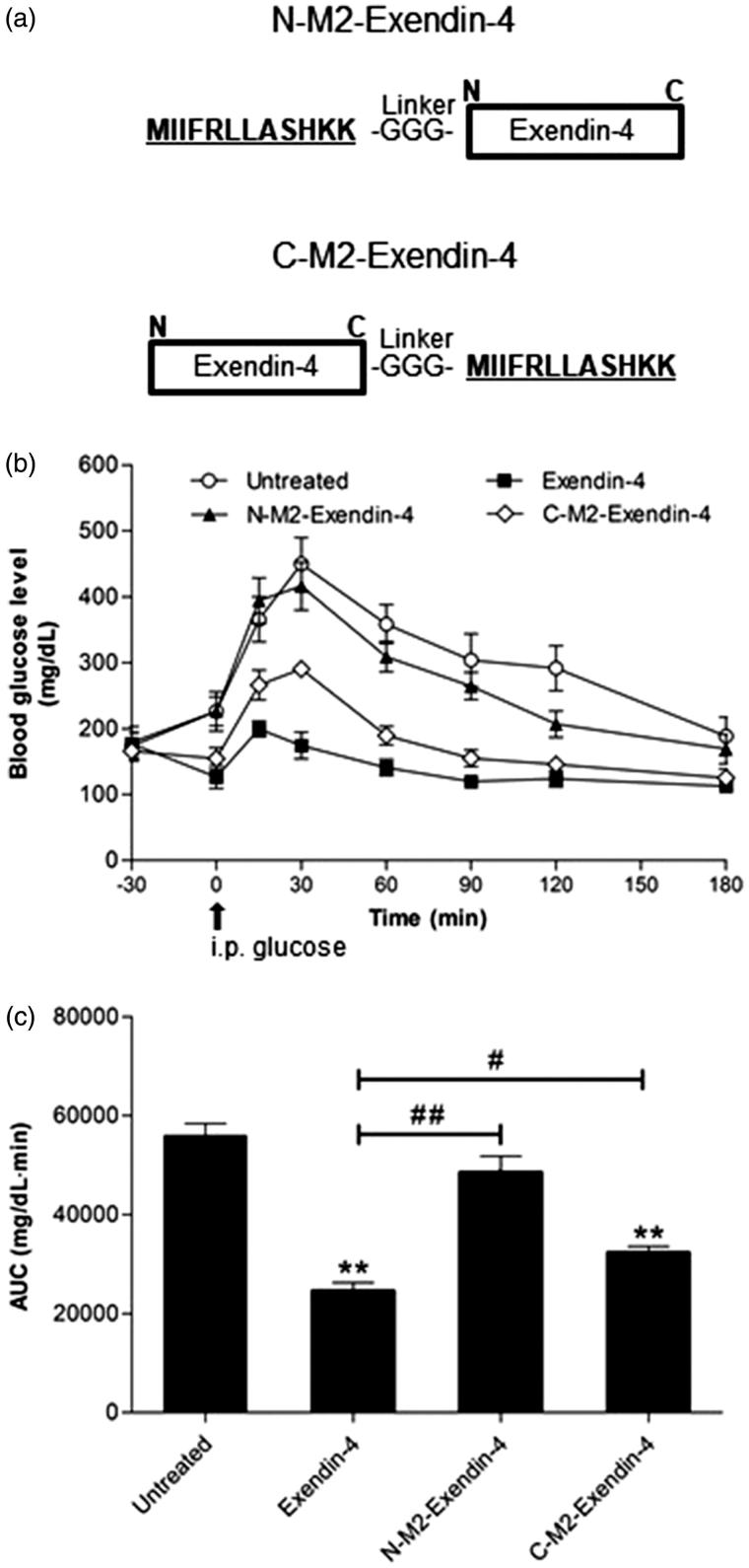
(a) Synthesis of chimeric PTD-exendin-4 peptides. TCTP-PTD 13M2 was attached to the N or C-terminus of exendin-4 directly through a peptide bond. (b) Hyperglycemic effect of exendin-4 and attachment of exendin-4 molecules to TCTP-PTD 13M2 in type 2 *db/db* mice. A single dose of either exendin-4 (2 μg/kg) or chimeric PTD-exendin-4 peptides (equivalent molar dosing) is administered to mice by s.c. injection. The time of glucose challenge in type 2 *db/db* mice is marked by the arrow. (c) Changes in the glucose response are expressed as the AUC. Vertical bars indicate means ± SEM (*n* = 6–8). **p < .01 compared with the untreated group; #*p* < .05 and ##*p* < .01.

Next, we examined the hypoglycemic effect of exendin-4 covalently linked with TCTP-PTD 13M2. We synthesized chimeric fusion peptides by attaching TCTP-PTD 13M2 to the N-terminus of exendin-4 (N-M2-Exendin-4) or C-terminus of exendin-4 (C-M2-Exendin-4) and introduced three glycine linker to minimize the steric hindrance between PTD and exendin-4 (Figure 3(a)). Thirty minutes prior to intraperitoneal administration of glucose, chimeric peptides were administered to *db/db* mice via subcutaneous injection to determine the hypoglycemic effect of exendin-4. We selected subcutaneous administration of exendin-4 alone as a positive control, because of its well-known hypoglycemic effect following subcutaneous injection. As shown in Figure 3(b), both linked peptides showed less hypoglycemic effect than exendin-4 alone, but subcutaneous injection of C-M2-Exendin-4 was more effective than that of N-M2-Exendin-4 (Figure 3(b)). The AUC of exendin-4 alone, N-M2-Exendin-4, and C-M2-Exendin-4 groups were 44.1, 86.8, and 57.8%, respectively (Figure 3(c)), suggesting that C-M2-Exendin-4 is more functional than N-M2-Exendin-4. In addition, the analysis of AUC results showed that hypoglycemic effect of C-M2-Exendin-4 was 42.2% better than that of the untreated group, although it did not reach the hypoglycemic effect of exendin-4 alone. This indicates that the biological activity of C-M2-Exendin-4 is maintained after the conjugation process using three glycine as a linker to avoid steric hindrance. However, it may be necessary to evaluate the hypoglycemic effects of products using longer linkers.

### Hypoglycemic effects of exendin-4 mixed with or covalently linked to TCTP-PTD 13M2

**Figure 4.**
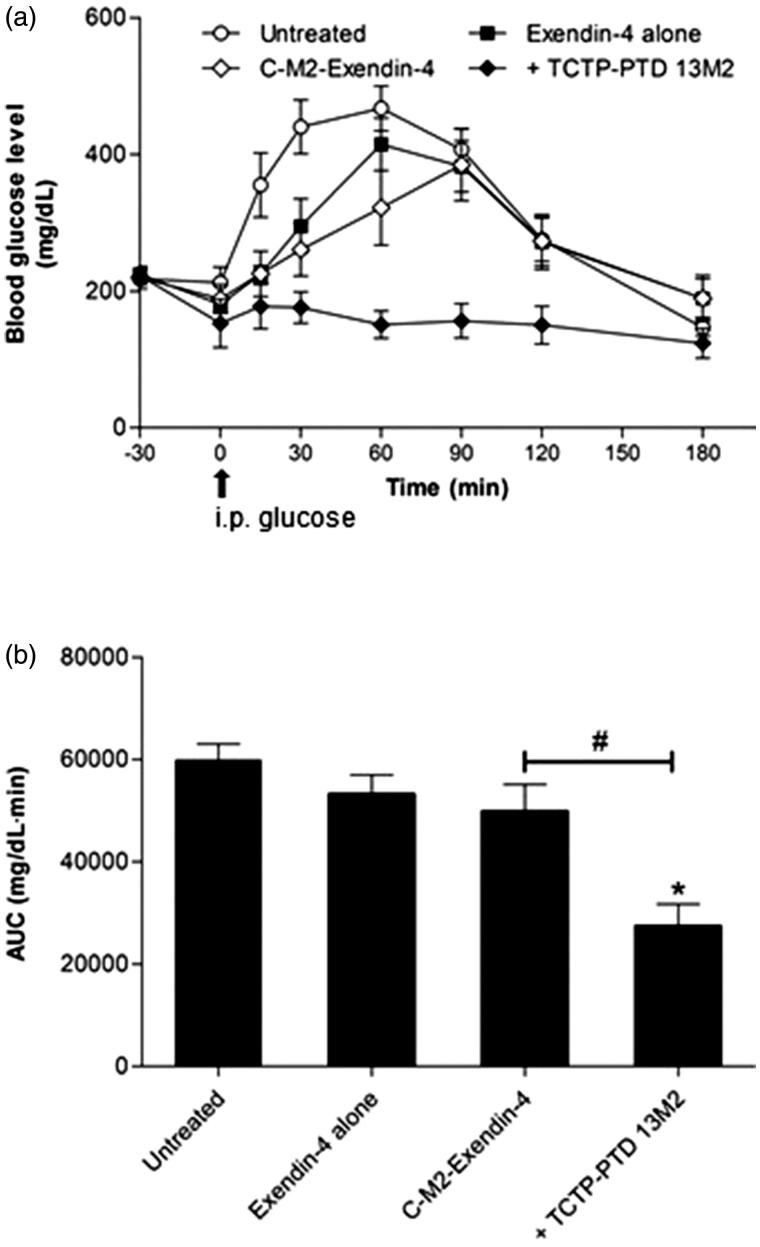
(a) Changes in blood glucose levels in type 2 *db/db* mice after nasal administration of exendin-4 alone, exendin-4 plus TCTP-PTD 13M2, and C-M2-Exendin-4 at a dose of 5 μg/kg (equivalent molar dosing). The time of glucose challenge in type 2 *db/db* mice is marked by the arrow. (b) Changes in the glucose response are expressed as the AUC. Vertical bars indicate means ± SEM (*n* = 6–8). *p < .05 compared with the exendin-4 alone; #*p* < .05.

We investigated the hypoglycemic effect of C-M2-Exendin-4 when intranasally administrated in *db/db* mice. It had little hypoglycemic effect, compared to that of the mixture of TCTP-PTD 13M2 and exendin-4 (Figure 4(a)). The AUC of C-M2-Exendin-4 intranasally administrated was similar to those of exendin-4 alone intranasally administrated and the untreated group (Figure 4(b)). These results suggest that exendin-4 directly attached to TCTP-PTD13M2 does not exert therapeutic effects when administered intranasally.

The hypoglycemic effect of C-M2-Exendin-4 subcutaneously administered was decreased by 42.2%, compared with the AUC of untreated group, whereas that of C-M2-Exendin-4 intranasally administered was insignificant, indicating that it is not successfully delivered or lost its biological activity during intranasal delivery in diabetic rats. The reason for this may be that there is a possibility of instability problem of the fusion peptide under the physiological buffer condition of the nasal cavity.

Most studies to date have used PTDs covalently linked to therapeutic cargoes or fusion proteins (Bolhassani et al., 2017). There have been reports that covalently linked form of PTD-cargo often interferes with the biological function of cargo (Kristensen et al., 2015). Co-administration of the PTD and cargo linked by noncovalent binding seems preferable because of the ease of the method, the automatic release of cargo in cells and tissues and maintenance of cargo’s biologic function. The studies demonstrated that factors such as cell type, concentration of PTDs and the nature of the cargo affect the function of cargoes (Jiang et al., 2014). Functions of the covalently or non-covalently bonded PTDs may vary from case to case (Bolhassani et al., 2017). Therefore, whether the cargo and PTD are chemically linked or simply mixed should be carefully decided by preliminary studies.

## Conclusions

Modification of TCTP-PTD 13 enhanced intranasal delivery of exendin-4. Among TCTP-PTD analogs TCTP-PTD 13M2 was the most effective for intranasal delivery of exendin-4 and showed enhanced hypoglycemic effect by 18.6% compared with that of exendin-4 plus TCTP-PTD 13, without any damage to nasal tissue. The conjugation of TCTP-PTD 13M2 and exendin-4, did not show hypoglycemic effect, when intranasally administered. In summary, nasal co-administration of TCTP-PTD 13M2 and exendin-4, which were mixed but not linked by covalent bond, showed the best hypoglycemic effect of exendin-4, like previously observed in intranasal delivery of insulin. These results suggest that the modified TCTP-PTD 13M2 might be universally used for nasal peptide/protein drug delivery. Further studies are required to resolve the delivery mechanism by TCTP-PTD analogs and some challenges for the use of these in clinical trials.
